# Association of induced abortion with preterm birth risk in first-time mothers

**DOI:** 10.1038/s41598-018-23695-7

**Published:** 2018-03-29

**Authors:** Li Ke, Weiyan Lin, Yangqi Liu, Weilin Ou, Zhifeng Lin

**Affiliations:** 1 Medical Record Department of The Third Affiliated Hospital of Guangzhou Medical University, Guangzhou, China; 2 Key Laboratory for Major Obstetric Diseases of Guangdong Provinces, Guangzhou, China; 3 Key Laboratory of Reproduction and Genetics of Guangdong Higher Education Institutes, Guangzhou, China

## Abstract

Women who have previously had an induced abortion (IA) before their first birth have been associated with preterm birth (PTB). However, previous studies on the PTB are inconsistent. Therefore, the aim of this study was to clarify the association between IA and PTB and low birth weight (LBW) for first-time mothers. A total of 3,684 Southern Chinese women who gave birth for the first time to a live singleton infants were recruited between January 2015 and December 2015 in the province of Guangdong, China. Univariable and multivariable analyses were conducted to determine whether IA was associated with PTB and LBW. Previous IA was not associated with increased risks of PTB or LBW, adjusted odds ratios were 0.80 (95% CI = 0.53 to 1.20) and 0.86 (95% CI = 0.57 to 1.31), respectively. Additionally, no significant associations were observed for infants born at before 37, before 32, and before 28 gestational weeks. And no significant associations were also observed for LBW measuring lower than 2500 grams and also measuring lower than 1500 grams. Our study suggested that a previous IA, as compared with women who reported no previous IA, does not increase the risk of PTB or LBW in subsequent pregnancy for the first-time mothers among Southern Chinese women.

## Introduction

Preterm birth (PTB), an important adverse pregnancy outcome, is one of the most important contributing factors to neonatal morbidity and mortality^1^. It affects approximately 14.9 million infants worldwide in 2010^2^. Despite recent advances in medical care, its relative importance is still increasing. At least 70% of perinatal mortality are attributable to newborns weighing less than 2500 grams^3,4^, of whom the majority are born preterm. It is well established that the occurrence and progression of PTB is a complicated process, which multiple genetic and environmental factors are involved in^5–9^. Thus, elucidation of the etiological factors for the development of preterm would be of great help to develop the effectively preventative and therapeutic approaches for neonatal morbidity and mortality.

Induced abortion (IA) is something many women undergo at some point in their lives. According to World Health Organization’s (WHO) statistics, 30% of pregnancies in Europe end in abortion, with the highest and lowest sub-regional termination rates world-wide being in Eastern and Western Europe at 43 and 12, respectively, per 1,000 fertile women^10^. In China, IA is widely practiced, about 8 million legally IAs are performed annually^11^. The average annual rate of IA was 28.95% among Chinese married women 20–49 years old, according to the data from 1979 to 2010^12^. IA not only does harm to the women^13^, particularly in adolescents and young women^14^, but also to the future child, e.g. PTB^15^ and low birth weight (LBW)^16^ children.

Previous studies have examined the relationship between IA (medical and surgical) and PTB, but an induced termination of pregnancy prior to the first birth adversely affecting the outcome of that birth has been previously debated^17–21^. There are evidences of an increased risk of PTB with many IAs prior to the first birth^17–20^. Nevertheless, some studies did not find an association between previous IA and PTB or LBW^21–24^. However, few studies have assessed the effect that IA have on the risk of PTB or LBW in a subsequent pregnancies for the first-time mothers among Southern Chinese women.

In view of the above controversies, we carried out a retrospective cohort study of 809 exposures with IA and 2875 non-exposures among Southern Chinese women to evaluate whether IA is associated with an increased risk of PTB or LBW in the subsequent pregnancy.

## Results

### Characteristics of the study subjects

The baseline maternal characteristics are shown in Table 1. IA was reported by 809 women, 588 of these reported one prior induced abortion, and the remaining 221 reported two or more prior IAs. There were 2,875 women who reported no prior IA. As expected, women who reported prior IA were slightly older compared with those with no prior IA (29.38 and 28.61 years old, respectively). Significant associations were also seen between health insurance, occupation, gravidity and education (all *P* < 0.01). However, no significant differences were observed for both groups with regard to BMI, gestational hypertension and caesarean section (all *P* > 0.05).

**Table 1:** Baseline maternal characteristics of the first-time mothers in the IA and non-IA groups.

Variable	Non-IA group (N = 2875)	IA group (N = 809)	*P*
Age,mean(SD)	28.61 (4.01)	29.38 (4.62)	<0.001
BMI (kg/m^2^)	20.66 (3.17)	20.86 (3.24)	0.111
Health insurance			<0.001
Care for urban employees	1952 (67.90)	478 (59.08)	
Free medical service	72 (2.50)	21 (2.60)	
Full-cost	851 (29.60)	310 (38.32)	
Occupation			<0.001
Professional	1337 (46.50)	316 (39.06)	
Business	282 (9.81)	101 (12.48)	
Housewife	320 (11.13)	115 (14.42)	
Others	936 (32.56)	277 (34.24)	
Gravidity			<0.001
1	2449 (85.18)	0 (0.00)	
2	307 (10.68)	508 (62.79)	
≧3	119 (4.14)	301 (37.21)	
Education			<0.001
Less then high school	377 (13.11)	148 (18.29)	
High school	344 (11.97)	138 (17.06)	
Cllege	2154 (74.92)	523 (64.65)	
Gestational hypertension			0.704
No	2771 (96.38)	782 (96.66)	
Yes	104 (3.62)	27 (3.34)	
Caesarean section			0.099
No	2034 (70.75)	548 (67.74)	
Yes	841 (29.25)	261 (32.26)	

Abbreviation: BMI, body mass index; IA, induced abortion.

### Association analysis between IA and PTB or LBW incidence risk

The associations between IA and the risk of PTB or LBW were presented in Table 2. Previous IA was not associated with increased risks of PTB or LBW, adjusted odds ratios (AOR) were 0.80 (95% CI = 0.53 to 1.20) and 0.86 (95% CI = 0.57 to 1.31), respectively. Moreover, we also categorized PTB separately for infants born at before 37, before 32 and before 28 gestational weeks, and no difference emerged (AOR = 0.83, 95% CI = 0.53 to 1.30; AOR = 0.64, 95% CI = 0.23 to 1.78; and AOR = 0.83, 95% CI = 0.09 to 7.55, respectively). Separately, no association was seen for infants with LBW less than 2500 grams and less than 1500 grams (AOR = 0.89, 95% CI = 0.56 to 1.43 and AOR = 0.77, 95% CI = 0.33 to 1.80, respectively).

**Table 2:** The association between IA and PTB or LBW in the IA and non-IA groups.

Variable	Non-IA group (N = 2875)	IA group (N = 809)	OR (95% CI)	*P*	AOR^*^ (95% CI)	*P*
PTB						
No	2628 (91.41)	733 (90.61)	1.00		1.00	
Yes	247 (8.59)	76 (9.39)	1.10 (0.84–1.44)	0.476	0.80 (0.53–1.20)	0.282
≧37 weeks	2628 (91.41)	733 (90.61)	1.00		1.00	
<37 weeks	198 (6.89)	62 (7.66)	1.12 (0.83–1.51)	0.445	0.83 (0.53–1.30)	0.411
<32 weeks	42 (1.46)	12 (1.48)	1.02 (0.54–1.96)	0.942	0.64 (0.23–1.78)	0.397
<28 weeks	7 (0.24)	2 (0.25)	1.02 (0.21–4.94)	0.976	0.83 (0.09–7.55)	0.868
LBW						
No	2625 (91.30)	741 (91.59)	1.00		1.00	
Yes	250 (8.70)	68 (8.41)	0.96 (0.73–1.28)	0.795	0.86 (0.57–1.31)	0.489
≧2500 grams	2625 (91.30)	741 (91.59)	1.00		1.00	
<2500 grams	196 (6.82)	50 (6.18)	0.90 (0.66–1.25)	0.537	0.89 (0.56–1.43)	0.633
<1500 grams	54 (1.88)	18 (2.22)	1.18 (0.69–2.03)	0.546	0.77 (0.33–1.80)	0.547

Abbreviations: OR, odds ratio; CI, confidence interval; AOR, adjusted odds ratio; PTB, preterm birth; LBW, low birth weight; IA, induced abortion.

^*^Adjusted OR and 95% CI were calculated by the logistic regression model after adjusting for age, health insurance, occupation, gravidity and education.

Additionally, women with two or more prior IA’s had a weakness significantly higher risk of delivering preterm in a subsequent pregnancy before adjusted confound factors (OR = 1.61, 95% CI = 1.07 to 2.43), but this difference disappeared after adjusting for confounding factors (AOR = 1.09, 95% CI = 0.53 to 2.22). Meanwhile, the AOR for LBW was 0.83 (95% CI = 0.54 to 1.27) for women with 1 IA and 1.73 (95% CI = 0.77 to 3.86) for women with 2 or more IAs (Table 3).

**Table 3:** The influence of prior IA on risk of PTB or LBW.

History of IA	PTB				LBW			
	No	Yes	OR (95% CI)	AOR^*^ (95% CI)	No	Yes	OR (95% CI)	AOR^*^ (95% CI)
0	2628 (91.41)	247 (8.59)	1.00	1.00	2625 (91.30)	250 (8.70)	1.00	1.00
1	541 (92.01)	47 (7.99)	0.92 (0.67–1.28)	0.77 (0.50–1.17)	547 (93.03)	41 (6.97)	0.79 (0.56–1.11)	0.83 (0.54–1.27)
≧2	192 (86.88)	29 (13.12)	1.61 (1.07–2.43)	1.09 (0.53–2.22)	194 (87.78)	27 (12.22)	1.46 (0.96–2.23)	1.73 (0.77–3.86)

Abbreviations: OR, odds ratio; CI, confidence interval; AOR, adjusted odds ratio; PTB, preterm birth; LBW, low birth weight; IA, induced abortion.

^*^Adjusted OR and 95% CI were calculated by the logistic regression model after adjusting for age, health insurance, occupation, gravidity and education.

## Discussion

In the present study, we conducted a retrospective cohort study in a Southern Chinese women to explore the association between IA, PTB, and LBW risk. Our results demonstrated that women with a previous IA were not associated with the risk of PTB or LBW incidence in a subsequent pregnancy for the first-time mothers.

The effect of an induced abortion causing preterm birth in a subsequent pregnancy is very controversial. In a review of risk factors for PTB, Hogue *et al*.^25^ included studies published concerning PTB subsequent to IA from the early 1960s to the early 1980s. Most these studies suggested that the relationship between IA and PTB failed to reach conventional levels of statistical significance. Conversely, in the review by Thorp *et al*.^26^, it was suggested that IA increased risk for PTB in subsequent pregnancy.

In recent years, various studies have addressed this association, but with inconsistent findings. There are evidences of an increased risk of PTB with an IA prior to the first birth^15,18,27–30^. However, some studies did not find an association between previous IA and PTB^24,31–36^. Furthermore, the relationship between IA and LBW is also debated, a cohort study by Denmark^30^ showed that IA was associated with the risk of LBW incidence in subsequent live births. Conversely, another study^18^ contradicted this result suggesting that IA did not elevated the risk for LBW.

However, the association of IA with PTB or LBW showed by our study was consistent with the previous publication by Chen *et al*.^31^, Chen compared pregnancy outcomes in women with one previous mifepristone IA with pregnancy outcomes in women with no previous IA and in women with one previous surgical IA. The study demonstrated that a previous medical IA was not associated with increased the risk of PTB or LBW incidence, as compared with no previous IA or a previous surgical IA. Moreover, Raatikainen and colleagues^24^ suggested that PTB was higher among women with one or more IA. Nevertheless, no such association emerged between IA and PTB after the adjustments for potential confounding factors. Additionally, a review study from Atrash *et al*.^22^. explored the risk in PTB and LBW after early surgical IA and reported no association between IA and a higher risk of PTB and LBW in the subsequent birth. Various studies performed since then are in line with such results^16,23,31,37^.

Although we controlled for several confounding factors known to be associated with adverse pregnancy outcomes, we did not adjust for variables such as alcohol consumption and maternal smoking since only one woman reported tobacco use in our data. Thus, we regarded these two variables not as confounders in our analysis.

However, the findings have been inconsistent. The inconsistency that emerged among the studies can at least partially be explained by differences in study design or power, by multiple comparisons, by recall bias and by differences in the methods used to perform IA at different times and in different countries. Additionally, epidemiological studies have been showed that revision of the uterine cavity might be responsible for endometritis, which is more likely to be the result of traumatic procedures^25,38^. This could also contribute to the differences in several studies. In spite of the research of PTB, the underlying etiology contributing to PTB needs to be further elucidated.

There are several advantages in our study as compared to Chen’s study^31^. First, the time of collecting the data was 17 years later than the time of the previous study, during which time the abortion laws and health and socioeconomic status of women had been changed significantly. These changes may have impacted the rate of IA and the recovery after performing an IA, which has a direct impact on the outcome of subsequent pregnancy. Second, in the present study, we not only controlled the possible influence of confounding variables by applying multivariate logistic regression analysis on a variety of risk factors that were found to be associated with PTB or LBW in previous studies, but also included other variables in our analysis, such as ‘health insurance’ and ‘gravidity’, which were not included in Chen’s study. Finally, the population we collected was from Southern China, whereas the population in Chen's study was from Northern China, which is also an innovation in our research. However, potential limitations of this study should be considered. One of the limitations of this study is lack of detailed information on types of PTB (i.e., spontaneous or iatrogenic PTB). We could not estimate the association between IAs and special types of PTB. Besides, some women might deliberately make inaccurate statements regarding previous IA, perhaps out of a perceived stigma associated with having had IA.

In summary, our study suggested that a previous IA, as compared with women who reported no previous IA, does not increase the risk of PTB or LBW in subsequent pregnancy for the first-time mothers among Southern Chinese women.

## Methods

### Study subjects

This retrospective cohort study was approved by the Medical Ethics Committee of the Third Affiliated Hospital of Guangzhou Medical University, Guangdong, China. All the methods in the present study were carried out in accordance with the approved guidelines. Between January 2015 and December 2015, a total of 3,684 women who had their first live birth at the Obstetric Department of The Third Affiliated Hospital of Guangzhou Medical University in Guangdong Province, China were included in the study.

The IA group (N = 809) consisted of women becoming mothers for the first time with a history of IA. Women pregnant with their first child and with no prior IA history were chosen for the non-IA group (N = 2,875). Women with prior deliveries, multifetal pregnancies, stillbirth, *in vitro* fertilization and embryo transfer were excluded from the study. For all subjects, the data were collected by interviewing women during their stay in the maternity unit, including maternal age, occupation, height and weight before of pregnancy, health insurance, education, gravidity and obstetric history. We derived the data on outcome measures, gestational week at birth (based on last menstruation and ultrasonography in early pregnancy), caesarean section and birth weight at delivery, from the hospital records. Gestational hypertension referred to diastolic pressure above 90 mmHg or systolic pressure above 140 mmHg on at least two occasions 24 hours apart, without proteinuria. Pre-pregnancy BMI, defined as the body weight in kilograms divided by the square of the height in metres (kg/m^2^), was obtained from the pre-pregnancy examinations at the first prenatal clinic visit, and all participants provided written informed consent.

Exposure was defined as the occurrence of a prior IA, including no previous IA (non-IA group), one previous IA or two or more previous IAs (IA group). For our analysis, we also categorized women who reported a prior IA as having had either one abortion or two or more abortions. Our primary outcome was PTB as birth before a 37-week gestation, we also categorized it as having occurred before 28 weeks, before 32 weeks and before 37 weeks. The secondary outcome was LBW that was defined as a birth weight less than 2500 grams at delivery, and very LBW was defined as infants weighing below 1500 grams.

### Statistical analysis

Differences in the distribution of demographic characteristics between the IA group and non-IA group were evaluated by using the *t* test (for continuous variables) or χ^2^ test (for categorical variables). The associations between IA and development of PTB or LBW were estimated using the adjusted odds ratio (OR) and their 95% confidence intervals (CI), as estimators of the relative risks of PTB or LBW, which were calculated in logistic regression models with adjustment for age, health insurance, occupation, gravidity and education. We also conducted an unconditional logistic regression to estimate the odds ratio of having a PTB or LBW with one previous IA and two or more prior IAs as compare to those who reported no previous IA. A *P* value of less than 0.05 was considered as statistically significant. All statistical analyses were conducted in the SAS 9.4 software (SAS Institute, Inc.,Cary, NC, USA).
